# The Human N-Alpha-Acetyltransferase 40 (hNaa40p/hNatD) Is Conserved from Yeast and N-Terminally Acetylates Histones H2A and H4

**DOI:** 10.1371/journal.pone.0024713

**Published:** 2011-09-15

**Authors:** Kristine Hole, Petra Van Damme, Monica Dalva, Henriette Aksnes, Nina Glomnes, Jan Erik Varhaug, Johan R. Lillehaug, Kris Gevaert, Thomas Arnesen

**Affiliations:** 1 Department of Molecular Biology, University of Bergen, Bergen, Norway; 2 Department of Surgical Sciences, University of Bergen, Bergen, Norway; 3 Department of Medical Protein Research, Vlaams Instituut voor Biotechnologie, Ghent, Belgium; 4 Department of Biochemistry, Ghent University, Ghent, Belgium; 5 Department of Surgery, Haukeland University Hospital, Bergen, Norway; Ludwig-Maximilians-Universität München, Germany

## Abstract

Protein N^α^-terminal acetylation (Nt-acetylation) is considered one of the most common protein modification in eukaryotes, and 80-90% of all soluble human proteins are modified in this way, with functional implications ranging from altered protein function and stability to translocation potency amongst others. Nt-acetylation is catalyzed by N-terminal acetyltransferases (NATs), and in yeast five NAT types are identified and denoted NatA-NatE. Higher eukaryotes additionally express NatF. Except for NatD, human orthologues for all yeast NATs are identified. yNatD is defined as the catalytic unit Naa40p (Nat4) which co-translationally Nt-acetylates histones H2A and H4.

In this study we identified and characterized hNaa40p/hNatD, the human orthologue of the yeast Naa40p. An *in vitro* proteome-derived peptide library Nt-acetylation assay indicated that recombinant hNaa40p acetylates N-termini starting with the consensus sequence Ser-Gly-Gly-Gly-Lys-, strongly resembling the N-termini of the human histones H2A and H4. This was confirmed as recombinant hNaa40p Nt-acetylated the oligopeptides derived from the N-termini of both histones. In contrast, a synthetically Nt-acetylated H4 N-terminal peptide with all lysines being non-acetylated, was not significantly acetylated by hNaa40p, indicating that hNaa40p catalyzed H4 N^α^-acetylation and not H4 lysine N^ε^-acetylation. Also, immunoprecipitated hNaa40p specifically Nt-acetylated H4 *in vitro*. Heterologous expression of hNaa40p in a yeast *naa40*-Δ strain restored Nt-acetylation of yeast histone H4, but not H2A *in vivo,* probably reflecting the fact that the N-terminal sequences of human H2A and H4 are highly similar to each other and to yeast H4 while the N-terminal sequence of yeast H2A differs. Thus, Naa40p seems to have co-evolved with the human H2A sequence. Finally, a partial co-sedimentation with ribosomes indicates that hNaa40p co-translationally acetylates H2A and H4. Combined, our results strongly suggest that human Naa40p/NatD is conserved from yeast. Thus, the NATs of all classes of N-terminally acetylated proteins in humans now appear to be accounted for.

## Introduction

N^α^-terminal acetylation (Nt-acetylation) occurs on 80–90% of soluble human proteins and 50–70% of soluble yeast proteins [1]–[3], making it one of the most widespread protein modifications in eukaryotes. This modification was previously largely believed to provide stability to proteins by preventing their degradation [4], which is contrary to the recent finding of Hwang *et al.* indicating that Nt-acetylation of specific N-termini in yeast acts as a degradation signal for the Doa10 ubiquitin ligase, thereby potentially targeting Nt-acetylated proteins for ubiquitin mediated degradation [5]. Studies over the last years have also linked Nt-acetylation to several other important protein functions, such as sub-cellular protein targeting [6]–[10]. Nt-acetylation of specific proteins is also essential for their correct activity or for their interaction with other proteins [11]–[13]. More recently, an inverse correlation between Nt-acetylation and the ability of a protein to post-translationally enter the secretory pathway was revealed [14]. Taken together, these studies implicate Nt-acetylation as an essential modulator of diverse protein properties [15]. Finally, a defective NAT was recently found to be the cause of a lethal syndrome affecting male infants, indicating that proper Nt-acetylation is functionally essential in humans [16].

Nt-acetylation is catalyzed by N-terminal acetyltransferases (NATs) and involves the transfer of the acetyl group from acetyl-coenzyme A to the primary α-amino group of the very N-terminal amino acid residue of a protein. The amino acid sequence at the N-terminus is the major factor determining whether or not a given protein is Nt-acetylated and by which NAT. In yeast, five different NAT types are identified, designated NatA-NatE [17]–[21]. All NATs are composed of a catalytical subunit responsible for catalyzing the acetylation reaction, and up to two auxiliary subunits. The NATs are associated with ribosomes where the acetylation reaction occurs cotranslationally as the peptide extrudes from the ribosome, typically after 20-50 residues have left the exit tunnel [22]–[24]. All NATs display by and large their own unique substrate specificity; NatA acetylates Alanine, Serine, Threonine, Glycine and Valine N-termini after initiator methionine (iMet) removal by methionine aminopeptidases [17], [18]. NatB acetylates proteins with Met-Asn-, Met-Asp- and Met-Glu- N-termini [25], while NatC acetylates the iMet when it is followed by a hydrophobic residue [26]. For NatE no substrates have been identified in yeast, and the yNatE deletion strain shows no obvious phenotype [23]. However, studies on homologues from higher eukaryotes show that this acetyltransferase has the potency to Nt-acetylate Met-Leu- and similarly starting peptide N-termini *in vitro* and is important for proper sister chromatid cohesion *in vivo* [27]–[31]. However, no *in vivo* substrates explaining this phenotype are identified. In addition to NatE, NatA, NatB, and NatC are also characterized in higher eukaryotes and shown to be evolutionarily conserved from yeast with respect to both complex composition and substrate specificity [1], [10], [32], [33]. Recently, an additional NAT exclusively present in higher eukaryotes was identified and designated Naa60/NatF. The acetyltransferase activity of hNatF partially explains the increased levels of Nt-acetylation seen in humans as compared to yeast [3].

The activity of Naa40p/NatD (Nat4) is so far characterized in yeast only. yNaa40p was identified in the search for the acetyltransferase responsible for Nt-acetylation of histone H4. In the same study, histone H2A was also identified as a substrate for yNaa40p [20]. Later, the activity of yNaa40p was denoted yNatD, and the enzyme was predicted to act co-translationally since a fraction of yNaa40p associated with ribosomes [34]. The N-terminal residue of histone H4 is a serine, hence a predicted NatA substrate. In contrast however to other NatA-type N-termini, Nt-acetylation of H4 was not affected in strains with mutant NatA subunits (*ard1^-^* or *nat1^-^*) suggesting that H4 is acetylated by another NAT [17]. The basic histone tail makes these proteins unusual substrates for a NAT since such residues are predicted to be inhibitory for acetylation [35]. In addition to perhaps acetylating only two selected substrates, H2A and H4, yNaa40p is also unusual in its requirement for as many as 20-50 available residues to effectively acetylate its substrates *in vivo* [34]. A search for additional substrates has proven unsuccessful, and this is also the case for the identification of putative auxiliary subunits which potentially remain to be identified. Song *et al*. failed to detect any phenotypes in a y*naa40*-deletion strain [20]. However, Polevoda *et al.* [34] later found that deletion of y*naa40* induced some minor growth defects when this deletion strain was grown on media containing an inhibitor of transcription (3-amino-1,2,4-triazole), anti-mitotic microtubule-destabilizing drugs (benomyl and thiabendazole) or a nonspecific membrane inhibitor (dinitrobenzene). A double mutant encoding histone H4 with K5R, K8R and K12R replacements, as to create an N^ε^-unacetylatable mutant, in addition to a disrupted y*naa40* gene, displayed an even greater growth defect on media containing some of the aforementioned compounds than either of the mutant strains alone, indicating that the lack of Nt-acetylation of histone H4 acts synergistically with the lack of lysine acetylation in the H4 tail, making Nt-acetylation of histone H4 part of a charge patch, thereby explaining the observed phenotypes based on a sub-optimal function of the histone tail [34].

The objective in the present study was to identify and investigate the human Naa40p/NatD. By the use of different methods, we found that the substrate specificity of hNaa40p is highly conserved from yeast, and recombinant hNaa40p was indeed capable of acetylating synthetic H4 and H2A peptides *in vitro*. Furthermore, heterologous hNaa40p also Nt-acetylated yeast H4 *in vivo*. Endogenous hNaa40p was additionally found to be associated with ribosomes in agreement with a co-translational activity, but interestingly a fraction of hNaa40p was also found to localize to the nucleus.

With the recent characterization of several human NATs and the current identification of the human NatD, it is likely that all human NATs acting co-translationally have been identified since all classes of substrates found to be Nt- acetylated are covered.

## Results and Discussion

### A human homologue of the yeast N-alpha acetyltransferase Naa40p (Nat4)

**Figure 1:**
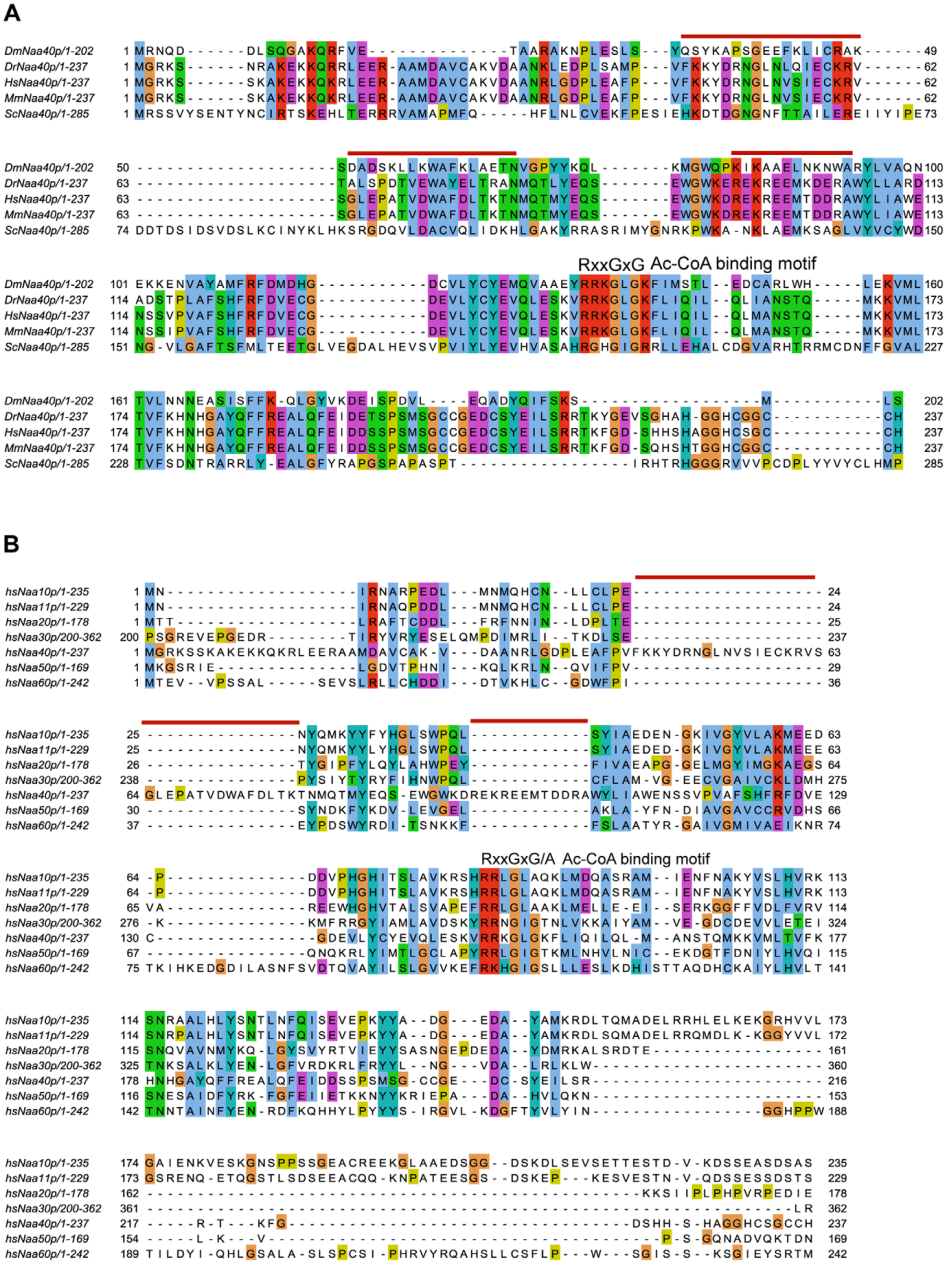
Amino acid alignments. (A) Amino acid sequence alignment of Naa40p from *Drosophila melanogaster* (Dm), *Danio rerio* (Dr), *Mus musculus* (Mm), *Homo sapiens* (Hs), and *Saccharomyces cerevisiae* (Sc). (B) Amino acid sequence alignment of hNaa40p and the catalytical subunit of NatA, NatB, NatC (residues 200–362), NatE and NatF. The conserved acetyl coenzyme A (Ac-CoA) binding motif RxxGxG/A, where x designates any amino acid, is indicated in both alignments. The red lines above the alignment represent examples of stretches of amino acid residues unique to Naa40p. Purple background indicates acidic residues, red indicates basic residues, orange indicates glycine, yellow indicates proline, blue indicates hydrophobic residues, green indicates polar residues, and turquoise indicates histidine and tyrosine residues. The alignments were created using Jalview multiple alignment editor [47], [48].

Sternglanz and coworkers previously identified yeast Naa40p (Nat4) to be an N^α^-terminal acetyltransferase, responsible for acetylating the N-termini of histones H2A and H4 [20]. In addition, they predicted yNaa40p to have homologues in higher eukaryotes, including humans. Later this putative human homologue (accession number NP_079047) was denoted hNaa40p, the catalytic subunit of the NatD type activity in accordance with the revised NAT nomenclature [19]. An alignment of the predicted Naa40 protein from different species is shown in Figure 1A. Of importance is the conservation of the acetyl-coenzyme A (Ac-CoA) binding motif, RxxGxG/A (where x can be any amino acid), from yeast to humans. This motif is found in all members of the Gcn5-related N-acetyltransferase (GNAT) superfamily and is probably required for Ac-CoA binding to the enzyme and hence its catalytic activity [36]-[38]. The conservation of this binding motif, in addition to an overall sequence similarity (23% amino acid sequence identity between yNaa40p and hNaa40p), is also seen in the alignment of the putative human Naa40p and the catalytical subunits of all characterized human NATs (Figure 1B), which also revealed that some stretches of amino acids are unique for Naa40p. These stretches are marked with red lines above the alignment. The conservation of the Ac-CoA binding motif is strongly indicative of an N-terminal acetyltransferase activity, and the alignment point to hNaa40p as being the yNaa40p orthologue.

### hNaa40p is a NAT selectively acetylating Ser-Gly or Gly-Gly starting peptides

**Figure 2:**
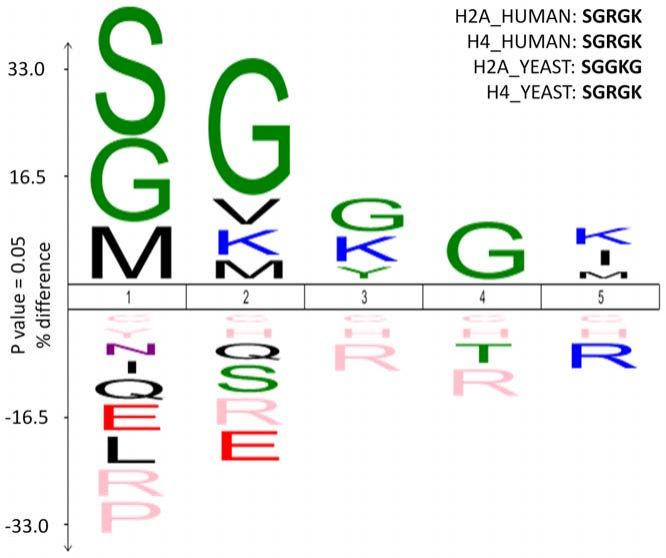
Sequence logos of the hNaa40p-specific substrate peptide sequences enriched from a N^α^-free-peptide library. An IceLogo of the 177 unique substrate peptides of recombinant hNaa40p is shown (255 substrate peptides in total). Numbering is such that Nt-acetylation occurs at position 0. Statistically significant residues with a p-value threshold of 0.05 or less are plotted. The amino acids heights are indicative for their degree of conservation at the indicated position. The frequency of the amino acid occurrence at each position in sequence set was compared with the human Swiss-Prot 56.0 database. Residues with statistically significant 0% frequency of occurrence as compared to Swiss-Prot are shown in pink. The N-terminal sequences of human and yeast histone H2A and H4 are indicated.

Although the amino acid sequence of hNaa40p clearly supports its candidacy as a novel human NAT of the NatD type, there is to our knowledge no experimental evidence yet in support of this. In order to assess whether hNaa40p is truly a NAT, the predicted h*NAA40* gene was cloned into a prokaryotic expression vector for recombinant MBP-hNaa40p fusion protein expression. A recently developed *in vitro* proteome-derived peptide library assay [30] was performed for an largely unbiased characterization of the potential enzymatic activity and substrate specificity of hNaa40p. As such, 177 unique substrate peptides were identified as being N-terminally acetylated by hNaa40p, providing evidence that hNaa40p is a genuine NAT. The resulting substrate specificity profile of hNaa40p, displayed in Figure 2, illustrates that hNaa40p preferentially acetylates Ser-Gly-Gly-Gly-Lys- starting peptides. The similarity between the sequence presented in the specificity profile and the N-terminal sequences of the human histones H2A and H4 being Ser-Gly-Arg-Gly-Lys- is striking, only deviating at position 3 with a Gly replaced by an Arg. It should be noted that since the assay is based on an N^ε^-modified proteome-derived peptide library generated by the use of trypsin, internal Arginines will be generally missed and consequently will be absent among preferred N-terminal residues. The 177 substrate peptides acetylated by hNaa40p in this screen range from 7-19 amino acid residues in length, a result in contrast with the finding of Polevoda *et al*. postulating that acetylation by yNaa40p required at least 30-50 amino acid residues at the N-terminus of histone H2A and H4 [34]. The apparent difference in requirement for more residues in the yeast experiments might implicate differences between species, or solely reflect different requirements between an *in vitro* setting with purified enzyme and peptides, and the *in vivo* setting in yeast where a ribosome-bound yNaa40p targets the emerging nascent polypeptide. For the *in vitro* assay described here, seven amino acids are clearly enough to ensure specificity and acetylation by hNaa40p. It should be noted that the N-termini in the peptide library assay do not represent true N-termini since they are generated by proteolytic cleavage. However, the significant match between the hNaa40p substrates from the specificity profile and the N-termini of histones H2A and H4 strongly suggests a conserved function from yeast to humans with respect to Nt-acetylation of histones H2A and H4.

### hNaa40p N-terminally acetylates histones H2A and H4 *in vitro*

**Figure 3:**
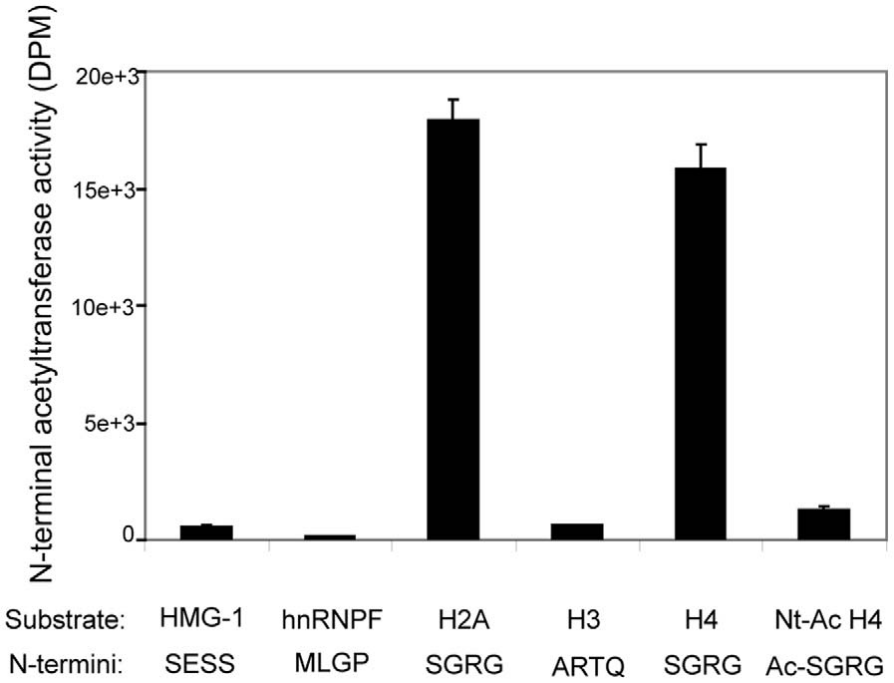
Recombinant MBP-hNaa40p acetylates H2A and H4 N-termini *in vitro*. MBP-hNaa40p was incubated with [1-^14^C]-Ac-CoA and selected peptides of 24–28 amino acids, representative of different protein N-termini. The first four N-terminal residues are shown for each substrate. The Nt-Ac-H4 substrate is a histone H4 peptide where the N-terminus is blocked by an acetyl group. Incorporated acetyl groups were measured as DPM. Error bars indicate standard deviation for at least three independent acetylation reactions.

In order to obtain further insight into the substrate specificity of hNaa40p, the quantitative aspect of substrate Nt-acetylation, and particularly the role of hNaa40p in histone acetylation thus evaluating its candidacy as a true orthologue of yeast Naa40p/NatD, an *in vitro* Nt- acetylation assay with purified recombinant hNaa40p was conducted. hNaa40p was challenged by synthetic peptides representing the N-termini of selected proteins, and interestingly, hNaa40p strongly preferred histones H2A and H4 (Figure 3). A histone H4 peptide with a synthetically pre-acetylated N-terminus was no longer acetylated efficiently by hNaa40p, consistent with hNaa40p acetylating the α-amino group of H4 and not significantly any of the lysine residues known to be N^ε^-acetylated by lysine acetyltransferases (KATs) [39]. Furthermore, only minor acetylation activity towards the HMG-1 (SESS), hnRNPF (MLGP), or histone H3 (ARTQ) peptides was observed. These three peptides are representatives of a classical NatA substrate [1], a NatE substrate [28], and a non-acetylated N-terminus [20], respectively. The lack of acetylation of the SESS peptide could be explained by the fact that a Glu residue in position P2′ is generally inhibitory for hNaa40p acetylation according to the specificity profile as deduced from the peptide library assays (Figure 2).

**Figure 4:**
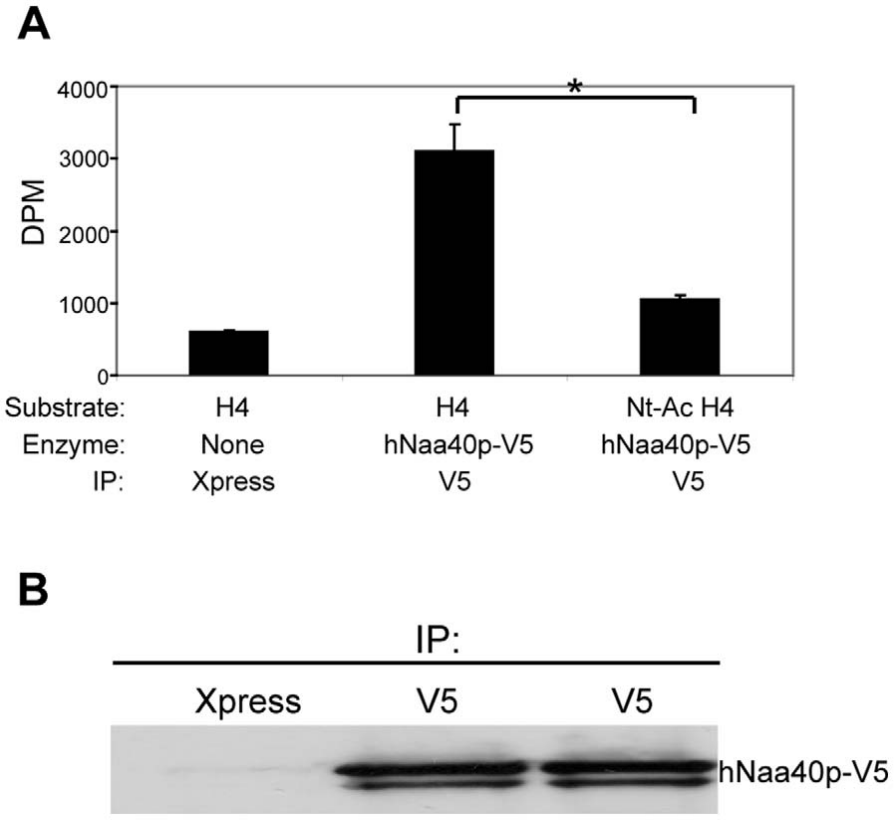
Immunoprecipitated hNaa40p-V5 acetylates histone H4 *in vitro.* (A) Immunoprecipitated hNaa40p-V5 acetylates histone H4 but not the N-terminally blocked histone H4 peptide (Nt-Ac-H4). Error bars indicate standard deviation for three independent acetylation reactions. Statistically significant results are indicated with * (p-value of 0.05 or less). The p-value (p = 0.0104) was calculated using Student's *t* test. (B) Following the acetylation assay, the agarose beads were subjected to SDS-PAGE and Western blotting using a specific antibody to the V5-tag.

To determine whether hNaa40p expressed in human cells possesses specific acetyltransferase activity, as shown when using recombinant hNaa40p purified from *E. coli*, hNaa40p-V5 was expressed in HEK293 cells and immunoprecipitated. Immunoprecipitated hNaa40p-V5 was used in an *in vitro* acetylation assay with synthetic peptides as described in Material and Methods. Histone H4 was significantly more acetylated as compared to the N-terminally blocked peptide (Figure 4). Taken together, the results from the *in vitro* experiments strongly suggest that hNaa40p is a NAT conserved from yeast which specifically Nt-acetylates histones H2A and H4 and that such specific acetylation may also take place in cultured cells.

### Ectopically expressed hNaa40p in yeast acetylates yeast histone H4 but not H2A *in vivo*

**Figure 5:**
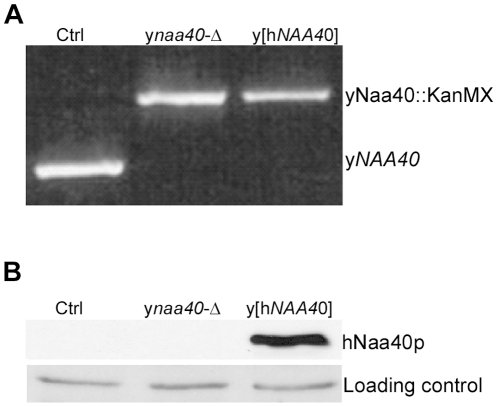
Generation of yeast strains. Three yeast strains were made by transformation; A normal yeast strain expressing normal levels of yNaa40p (Ctrl). A strain where the y*NAA40* gene was disrupted by insertion of a kanMX cassette (y*naa40*-Δ), and a y*naa40* deletion strain ectopically expressing h*NAA40* (y[h*NAA40*]). (A) Disruption of *NAA40* was verified by isolation of genomic DNA, and PCR using *NAA40-*specific primers. (B) Western Blotting using a specific anti-hNaa40p antibody confirmed the expression of hNaa40p in the y[h*NAA40*] strain.

**Figure 6:**
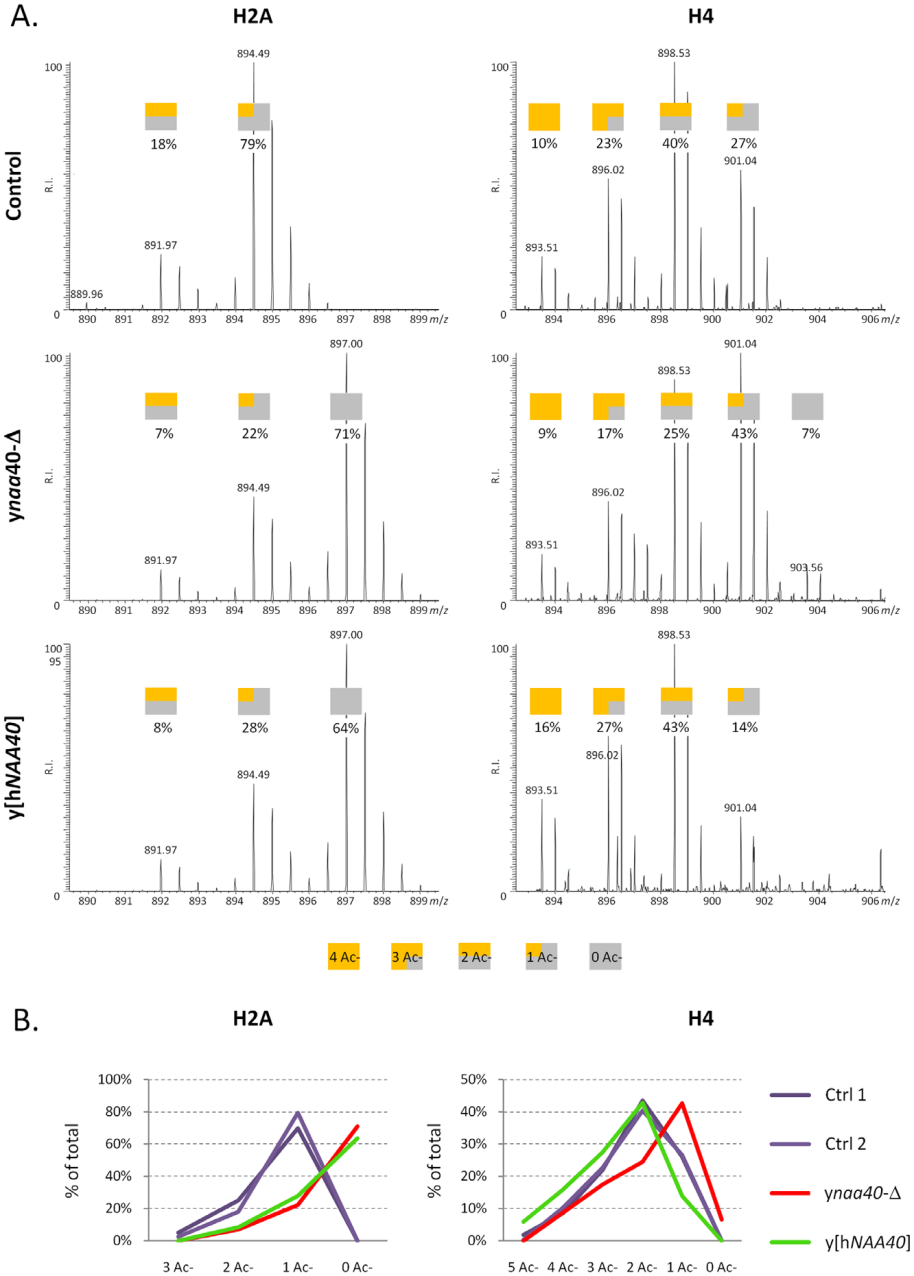
Ectopically expressed hNaa40p in yeast acetylates histone H4 but not H2A *in vivo*. (A) The mature N-terminus of yeast histone H2A (^2^SGGKGGKAGSAAKASQSR^19^) (left panels) and H4 (^2^SGRGKGGKGLGKGGAKR^18^) (right panels) was identified as being partially *in vivo* N-acetylated. Various N-acetylated forms of these histone N-termini were detected in all samples analyzed. A decrease of 5 Da, corresponds with the gain of an extra *in vivo* N-acetyl group (i.e. all free amines were *in vitro* N-acetylated using ^13^C_2_D_3_-NHS acetate; i.e. adding a 5 Da heavier acetyl group to free amines). Colored squares indicate the number of *in vivo* (orange) and *in vitro* (gray) acetyl group present per detected isotopic envelope. Clearly deletion of y*NAA40* reduces the overall degree of N-acetylation (middle panels) for both histone N-termini while the ectopic expression of hNaa40p restores and even augments the degree of N-aectylation of the H2A N-terminus, while not having any observable effect on the overall N-acetylation status of the H4 N-terminus (B) The percentage of all isobaric N-terminal variants as shown in panel A were calculated and their contribution to the overall percentage of the respective histone N-terminus plotted.

In order to assess whether hNaa40p represents a true orthologue of yNaa40p *in vivo*, we generated a yeast *naa40*-Δ strain expressing hNaa40p (Figure 5). When comparing Nt-acetylation of histones in control yeast (yeast Ctrl 1 or 2) to yeast lacking *NAA40* (y*naa40*-Δ) and to the yeast strain lacking *NAA40* but expressing hNaa40p (y[hNAA40]), hNaa40p expression restored the diminished histone H4 acetylation (Figure 6). In fact, the expression of hNaa40p increased the total level of H4-acetylation (*i.e*. the combined Nt-acetylation and lysine acetylation observed). This could indicate that Nt-acetylation stimulates lysine acetylation *in vivo,* that hNaa40p is also capable of catalyzing lysine acetylation of histone H4, or perhaps that the degree of Nt-acetylation is higher in yeast expressing hNaa40p as compared to yNaa40p. Lysine acetylation is normally catalyzed by the lysine acetyltransferases (KATs), while Nt-acetylation is catalyzed by NATs. However, a dual NAT/KAT-role for the NAT-enzymes has been suggested for hNaa50p [28], [40] as well as for hNaa10p [41], [42]. The *in vitro* assays described above (Figures 3 and 4) indicate that hNaa40p mostly acts as a NAT towards histone H4, but a minor KAT activity might occur. Further detailed experiments are required to conclude on this point. As expected, no alterations in histone acetylation were observed for H2B and H3 for any of the yeast strains used (data not shown). Surprisingly, histone H2A, which was less acetylated in the yeast *naa40-*Δ strain as compared to the controls, was not acetylated by hNaa40p (Figure 6). This probably reflects the fact that the first five N-terminal amino acid residues of histone H2A are altered from yeast (Ser-Gly-Gly-Lys-Gly-) to humans (Ser-Gly-**Arg**-**Gly**-**Lys**-) to become identical to the Nt-sequences of yeast and human histone H4 (Ser-Gly-Arg-Gly-Lys-). From the *in vitro* specificity profile (Figure 2), it is clear that Lys4 and Gly5 are not optimal for hNaa40p activity. Naa40p might have evolved to become even more specific in humans, acetylating the N-termini of the very similar human H2A and H4, while the yeast homologue is able to Nt-acetylate the more divergent N-termini of yeast H2A and H4. Despite the lack of yeast histone H2A acetylation, the *in vitro* and *in vivo* data combined, strongly suggest that hNaa40p is the true orthologue of yNaa40p.

### Subcellular localization of hNaa40p

**Figure 7:**
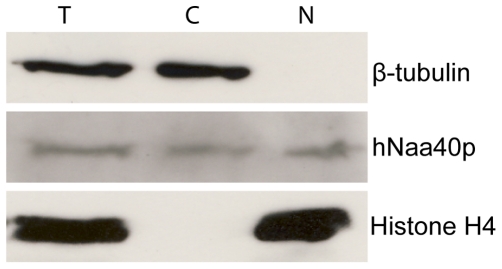
Endogenous hNaa40p localizes to the cytosol and nucleus. A431 cells were harvested and fractionated. Total cell lysate (T), cytosolic fraction (C), and nuclear fraction (N) were analyzed by SDS-PAGE and western blotting. Beta-tubulin was used as a cytosolic marker, while histone H4 was used as a nuclear marker. hNaa40p was detected in all fractions using an anti-hNaa40p antibody. Results shown are representative of three independent experiments.

Subcellular fractionation was performed to elucidate on the localization pattern of hNaa40p. As shown in Figure 7 endogenous hNaa40p localizes to both the cytoplasm and the nuclei of A431 cells. The same localization was also observed in HEK293 cells (data not shown).

**Figure 8:**
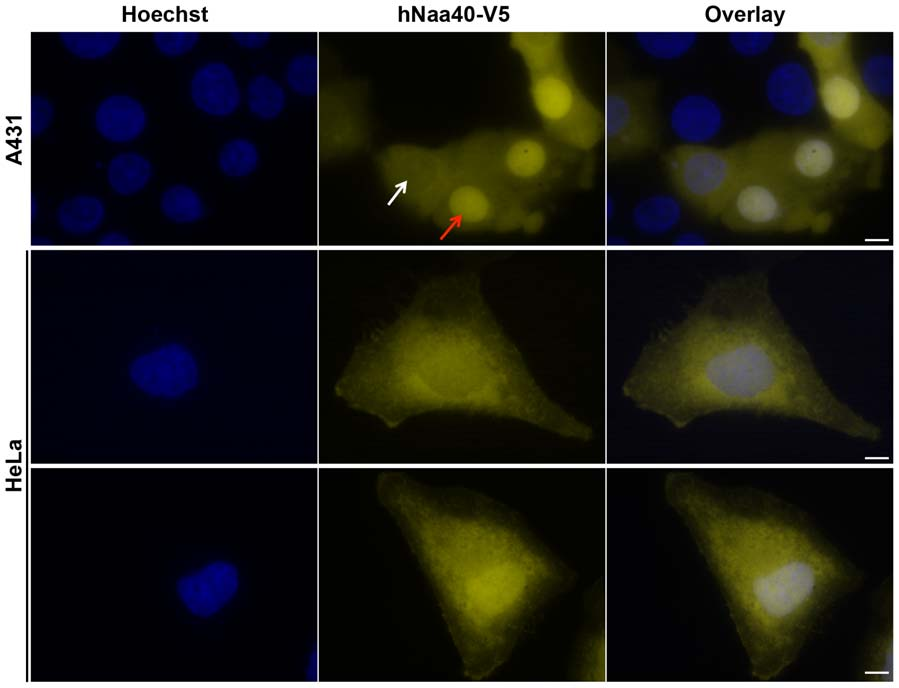
Subcellular localization of hNaa40p-V5. A431 cells or HeLa cells were transfected with plasmid encoding hNaa40p-V5. The V5-tagged hNaa40 protein was visualized using mouse IgG_2a_ Anti-V5 and Alexa-594 conjugated anti-mouse IgG_2a_ monoclonal antibody. Hoechst (33342) staining was used to visualize the cell nuclei. hNaa40p-V5 displayed a distributed cytoplasmic localization with some degree of nuclear localization. Note that the degree of nuclear localization varied between individual cells in the same sample, some expressing hNaa40p mainly in the cytosol and to a lesser degree in the nucleus (Panel 1 (white arrow, and 2); and others showing hNaa40p enriched in the nucleus (Panel 1 (red arrow), and 3). Scale bars are 5 µm.

In addition, subcellular localization of exogenously expressed hNaa40p-V5 was determined using immunofluorescence microscopy (Figure 8). In both cell lines investigated, HeLa and A431, hNaa40p-V5 was found to have a distributed cytoplasmic localization with some degree of nuclear localization. Surprisingly, two main populations of localization patterns were observed, one where hNaa40p-V5 was found mainly in the cytoplasm and to a lesser degree in the nucleus (Figure 8, panel 1 (white arrow), and 2), and another where hNaa40p-V5 was found enriched in the nucleus (Figure 8, panel 1 (red arrow), and 3). The latter could possibly be due to an artificial nuclear accumulation of the overexpressed protein. However, only low to medium intensity cells were taken into account. In addition, cells with a low amount of hNaa40p-V5 in the nucleus compared to the cytoplasm, were also observed among highly overexpressing cells (figure S1). Furthermore, there also existed a smaller population of cells in which the fluorescence intensity level was equal in the two compartments (data not shown), potentially representing an intermediate or transition stage.

### hNaa40p associates with ribosomes

**Figure 9:**
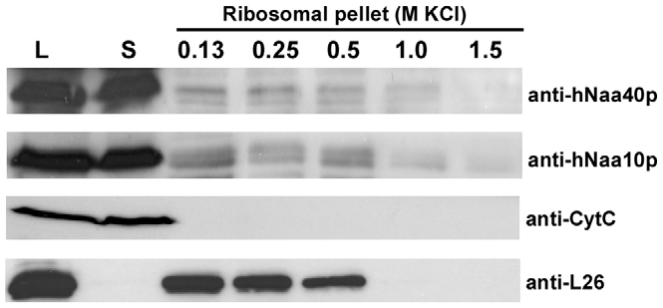
A fraction of hNaa40p co-sediments with polysomes . Polysomal pellets from HEK-293 cells were resuspended in buffer containing increasing concentrations of KCl. Cell lysate (L), supernatant post first ultracentrifugation (S) and polysomal pellets after KCl treatment were analyzed by SDS-PAGE and Western blotting. The membrane was incubated with anti-hNaa40p, anti-hNaa10p, anti-L26 (ribosomal protein), and anti-CytC antibodies. Results shown are representative of three independent experiments.

NATs are involved in co-translational acetylation of nascent polypeptides, and are therefore physically associated with ribosomes. The yeast Naa40p was also demonstrated to associate with ribosomes [34]. In order to investigate whether hNaa40p shares this feature, ribosomes from HEK293 cells were isolated and the binding of endogenous hNaa40p was analyzed by Western blotting. Indeed, a small but reproducible fraction of hNaa40p was detected in the polysomal pellet while the majority of hNaa40p was present in the supernatant post ultracentrifugation (Figure 9). As demonstrated here for the catalytic subunit of the NatA complex, hNaa10p, this pattern is similar to what was observed previously for all the other human NAT subunits [10], [30], [33], [43]. yNaa10p has previously been shown to associate with ribosomes through an interaction with the auxiliary subunit yNaa15p [23]. No such auxiliary subunit has been identified for Naa40p neither in yeast nor in humans, suggesting that hNaa40p itself might interact with the ribosome directly. However, the lack of identified additional NatD subunit(s) does not exclude their presence. The large fraction of hNaa40p not in complex with ribosomes indicates that most of hNaa40p is in a free form in the cytoplasm or in the nucleus based on the fractionation and immunofluorescence experiments presented above.

### Concluding remarks

In conclusion, we have here identified the human Naa40p (NatD) acting as a co-translational NAT towards the N-termini of the histones H2A and H4. Overall, the set of functionally characterized human NATs now comprises NatA-NatF which are likely to be responsible for all co-translational Nt-acetylation events in human cells. A potential role for hNaa40p in the nucleus as well as a potential function as a KAT awaits further investigation.

## Materials and Methods

### Construction of plasmids

The plasmid (ph*NAA40*-V5) encoding V5-tagged hNaa40p was constructed from cDNA made from total RNA isolated from human HEK293 cells. Superscript™II Reverse Transcriptase (Invitrogen) was used to make cDNA. PCR products were inserted into the TOPO TA vector pcDNA 3.1/V5-His© TOPO® (Invitrogen) according to the instruction manual. Prokaryotic expression vector for h*NAA40* was made by subcloning the amplified h*NAA40* gene from ph*NAA40-V5* to the pETM41 expression vector using XmaI and EcoRI restriction sites. pETM41 was generously provided by G. Stier, EMBL, Heidelberg.

### *E. coli* hNaa40p expression and purification

*E. coli* BL21 Star™ (DE3) (Invitrogen) was transformed by heat shock with the pETM41-h*NAA40* plasmid encoding a MBP-hNaa40p fusion protein, and grown overnight on LB agar containing kanamycin. A well separated colony was picked and inoculated at 37°C in 200 ml LB medium supplemented with kanamycin to an OD_600_ of 0.6. Protein expression was induced by IPTG (1 mM) at 20°C for 18 hours. The culture was harvested by centrifugation at 3000 x *g* for 20 minutes. The cells were lysed by sonication in lysis buffer (50 mM Tris-HCl (pH 8.3), 300 mM NaCl, 1 mM DTT, 1 tablet EDTA-free protease Inhibitor cocktail per 50 ml (Roche)) and cleared by centrifugation at 10000 x *g* for 20 minutes. Cleared lysate was applied on a metal affinity FPLC column (HisTrap HP, GE Healthcare, Uppsala, Sweden), and MBP-hNaa40p was eluted with 300 mM imidazole in 50 mM Tris (pH 8.3), 300 mM NaCl, 1 mM DTT. MBP-hNaa40p was further purified on a size exclusion chromatography column (Superdex™ 75, GE Healthcare).

### *In vitro* proteome-derived peptide library Nt-acetylation assay

Proteome-derived N^α^-free peptide libraries were generated from human K-562 cells as described previously [30]. 100 nmol of the desalted N^α^-free peptide pool was reconstituted in acetylation buffer (50 mM Tris-HCl (pH 8.3), 1 mM DTT, 800 µM EDTA, 10% glycerol) together with equimolar amounts of a stable isotope encoded variant of acetyl-CoA, ^13^C_2_-acetyl CoA, (99% ^13^C_2_-acetyl CoA, ISOTEC-Sigma (lithium salt)) and 500 pmol of enzyme (i.e. recombinant MBP-hNaa40p) was added to a final reaction volume of 500 µl. The reaction was allowed to proceed for 2 h at 37°C and stopped by addition of acetic acid to a 5% final concentration. NAT oligopeptide-substrate recovery and LC-MS/MS analyses was performed as decribed previously [30].

### LC-MS/MS analysis

LC-MS/MS analysis was performed as described previously [30]. The generated MS/MS peak lists were then searched with Mascot using the Mascot Daemon interface (version 2.2.0, Matrix Science). Searches were performed in the Swiss-Prot database with taxonomy set to human (H*omo sapiens*) (UniProtKB/Swiss-Prot database). Trideutero(d_3_)-acetylation at lysines, carbamidomethylation of cysteine and methionine oxidation to methionine-sulfoxide were set as fixed modifications. Variable modifications included ^13^C_2_-acetylation, d_3_-acetylation, acetylation and pyroglutamate formation of N-terminal glutamine. Endoproteinase Arg-C/P (Arg-C specificity with arginine-proline cleavage allowed) was set as enzyme allowing no missed cleavages. The mass tolerance on the precursor ion was set to 10 ppm and on fragment ions to 0.5 Da. Peptide identifications were considered significant when their Mascot ion score was above the identity threshold set at a 99% confidence interval and when they were top-ranked.

### *In vitro* acetylation assay using synthetic oligopeptides as substrates

For the *in vitro* acetylation assay using custom-made synthetic peptides, purified MBP-hNaa40p (400 nM) was mixed with selected oligopeptide substrates (200 µM) and 200 µM of [1-^14^C] acetyl-CoA in a total volume of 170 µl acetylation buffer (50 mM Tris-HCl (pH 8.3), 800 µM EDTA, 10% glycerol, 1 mM DTT) and incubated for 1 hour at 37°C. Following incubation, 150 µl SP Sepharose beads (50% slurry with 0.5 M acetic acid) were added to the mix and incubated at room temperature for 5 minutes. After centrifugation 6000 x *g* for 2 min, the beads were washed three times with 0.5 M acetic acid. Following the last wash, the supernatant was removed and the beads washed with 300 µl ice cold methanol, after centrifugation the supernatant was removed and the beads were transferred to 5 ml counting fluid and radioactivity was measured as disintegrations per minute (DPM) using a scintillation counter.

For the immunoprecipitation experiments, HEK293 cells were transfected with a plasmid encoding hNaa40p-V5 using Fugene® 6 Transfection reagent. Cells were harvested 48 hours post-transfection and resuspended in lysis buffer (50 mM Tris-HCl (pH 7.5), 150 mM NaCl, 5 mM EDTA, 0.5% NP-40, Pefabloc). After lysis on ice for five minutes, the lysate was cleared by centrifugation 3000 x *g* for 5 minutes. hNaa40p-V5 was immunoprecipitated from the lysate using specific antibodies against the V5-tag (Invitrogen) and Protein A/G Agarose beads (Santa Cruz). Immunoprecipitation using antibodies against the Xpress-tag (Invitrogen) was used as a negative control. Following washing (3 times) with lysis buffer and acetylation buffer the immunoprecipitates were used in an *in vitro* acetylation assay as described above.

### Yeast strain generation and cultivation for histone isolation

Yeast was grown and manipulated according to standard procedures [44]. Expression vector for h*NAA40* was made by subcloning the amplified h*NAA40* gene from ph*NAA40*-V5 to pBEVY-U bidirectional expression vector [45] using the BsmBI restriction site.

The *S. cerevisiae* MAT alpha strain Y10000 was transformed with pBEVY-U and acted as control strains (yeast Ctrl). Strain Y16202 (*naa40*-Δ, Euroscarf) was transformed with pBEVY-U or pBEVY-U-h*NAA40* to generate the strains y*naa40*-Δ and y[h*NAA40*], respectively. The three strains generated were selected on plates lacking uracil. Disruption of *NAA40* was verified by isolation of genomic DNA, and PCR using the primers yNAA40-86F: 5′-CCACATATGTATGCGCGGTGC-3′ and yNAA40 941R: 5′- GGAGTCAAGGATTCGAGTCGC-3′. Expression of hNaa40p was verified by SDS-PAGE and Western blotting analysis of yeast lysates using anti-hNaa40p (Biosite).

For histone isolation, the strains were cultivated in 10 ml synthetic medium lacking uracil (Sigma) to an OD600 nm of ∼3. Cells were collected by centrifugation for 5 min at 1500 x *g* and resuspended in 500 µl lysis buffer (0.2% TRITON-X-100, 10 mM HEPES (pH 7.6) 1.5 mM MgCl_2_, 10 mM KCl, 1 mM Pefabloc, 1 mM Na_3_VO_4_ and 5 µM TSA). Glass beads were added before several rounds of vortex/ice. After lysis, the glass beads were removed and the supernatant was centrifuged for 5 minutes at 3000 x *g.* Pellets were washed in lysis buffer followed by centrifugation for 5 min at 300 x *g*. The pellets were incubated in 0.4 M HCl for 1 hour and centrifuged for 15 min at 13000 x *g*. 6 volumes of ice-cold aceton were added to the supernatant and acid-extracted proteins precipitated overnight at −20°C. After centrifugation for 15 min at 13000 x *g,* and 3 rounds of washing with ice-cold aceton the pellets were dried for 5 min in a vacuum concentrator.

### SDS-PAGE of histone samples and determination of the N-acetylation status of yeast histone N-termini using in-gel stable-isotope labeling (ISIL) followed by in-gel digestion

All dried histone pellets were dissolved in 50 µl sample buffer (8 M urea, 5% β-mercaptoethanol and 10 mM Tris-HCl (pH 7.0)). 20 µl of each sample was added SDS-sample buffer (Biorad XT sample buffer) and the samples were analyzed on a 4%-16% gradient XT precast Criterion gel using XT-MOPS buffer (Biorad).

In-gel-stable isotope labelling was performed as described previously [43] with the following modifications; primary amines were (^13^C_2_d_3_)-acetylated by the addition of 1 mg N-hydroxysuccinimide (^13^C_2_d_3_)-acetate and trypsin digestion proceeded for 30′ at 37°C, representative of a sub-optimal incubation time, to increase the occurrence of missed cleavages (i.e. the yeast histone H4 N-terminus harbours an Arg-residue at the third position (i.e. the N-terminus w/o the iMet)) thereby allowing for the identification of the yeast H4 histone N-terminus (next to the H2A N-terminus).

The resulting peptide mixtures were acidified (0.1% formic acid) and analyzed by LC-MS/MS analysis as described previously [43]. The generated MS/MS peak lists were then searched with Mascot using the Mascot Daemon interface (version 2.2.0, Matrix Science). Searches were performed in the Swiss-Prot database with taxonomy set to human (*Homo sapiens*) (UniProtKB/Swiss-Prot database). ^13^C_2_d_3_- or normal acetylation at lysines, ^13^C_2_d_3_- or normal acetylation of the N-terminus, pyro-glutamate formation of N-terminal glutamine and methionine oxidation to methionine-sulfoxide were set as variable modifications. Endoproteinase Arg-C/P (Arg-C specificity with arginine-proline cleavage allowed) was set as enzyme allowing 2 missed cleavages. The mass tolerance on the precursor ion was set to 10 ppm and on fragment ions to 0.5 Da. Peptide identifications were considered significant when their Mascot ion score was above the identity threshold set at a 99% confidence interval and when they were top-ranked.

The extent of histone N-acetylation was calculated from the peptide ion signals observed in the MS spectra as described in [3].

### Cell culture

HEK293 cells (human embryonic kidney 293 cells; A.T.C.C. no. CRL-1573), HeLa cells (epithelial cervix adenocarcinoma; A.T.C.C. no. CCL-2), and A431 cells (human epidermoid carcinoma cells A.T.C.C. no. CRL-1555) were cultured in 5% CO_2_ at 37°C in Dulbecco's modified Eagle's medium (DMEM) supplemented with 10% heat inactivated fetal bovine serum (FBS), and 3% L-glutamine.

### Immunofluorescence

For fluorescent microscopic analysis, cells were transfected with plasmid encoding hNaa40p-V5. Transfection was carried out using Fugene® 6 Transfection reagent (Roche) and 0.5 µg plasmid per ml medium. 24 hours post transfection, cells were incubated for 10 min with Hoechst 33342 (Invitrogen) (0.4 µl per ml medium). Cells were then washed in PBS and fixed for 20 min in 4% paraformaldehyde in PBS. Fixed cells were washed three times in PBS, permeabilized using 0.2% Triton X-100 for 10 min and then washed once in PBS before incubation in blocking buffer (10% BSA) for 1 hour. Primary antibody, Mouse IgG2a anti-V5 (Invitrogen), diluted 1∶200 in 2% BSA was incubated for 1 h. Cells were washed one time in 0.1% Triton X-100 for 5 min, followed by two washes in PBS. The fluorochrome-conjugated secondary antibody, Alexa Fluor® 594 goat anti-mouse IgG_2a_ (Molecular Probes, Eugene, Oregon, USA), diluted 1∶100 in 2% BSA was incubated for 40 min. Cells were again washed once in 0.1% Triton X-100 for 5 min followed by two PBS washes. Finally, coverslips were dipped in ddH_2_O before mounting on glass slides using SlowFade Gold antifade reagent (Invitrogen) after which coverslips were sealed. Imaging was performed using a Leica DMRXA microscope equipped with a 2× magnification lens, a Leica DC500 camera, and a Leica HCX PL APO 100× oil immersion objective. Hoechst stain was visualized using a 340–380 nm bandpass filter with longpass suppression filter (425 nm); Alexa-594 was visualized using a 560/40 nm bandpass filter with bandpass suppression filter (645/75 nm). Untransfected cells and secondary antibody staining only were considered as a control for unspecific binding of the antibodies.

### Subcellular fractionation

Subcellular fractionation was used to separate nuclear from cytosolic proteins. Based on the protocol by Suzuki et al. [46], cells were washed in PBS and harvested by “pop spin” for 10 seconds (3000 x *g*). The resulting cell pellet was resuspended in 900 µl of ice-cold lysis buffer (0.1% NP40 in PBS) and a sample was taken as total lysate fraction. Following a second pop-spin for 10 seconds, a sample from the supernatant was removed representative of the cytosolic fraction. The nuclear pellet was washed by resuspension in 1 ml lysis buffer followed by centrifugation for 10 seconds. The supernatant was discarded and the pellet was resuspended in sample buffer and designated as the nuclear fraction. The nuclear fraction and the total lysate fraction were sonicated two times for 10 seconds. The subcellular fractions were analysed by SDS-PAGE and Western blotting.

### Ribosome isolation

Approximately 2×10^7^ HEK293 cells were used per experiment. Prior to harvesting, cells were treated with 10 µg/ml cycloheximide (CHX) for 5 minutes at 37°C. Cells were harvested, lysed with KCl-containing ribosome lysis buffer (1.1% (w/v) KCl, 0.15% (w/v) triethanolamine, 0.1% (w/v) magnesium acetate, 8.6% (w/v) sucrose, 0.05% (w/v) sodium deoxycholate, 0.5% (v/v) Triton-X100, 0.25% (v/v) Pefabloc), and incubated on ice for 15 minutes. After removing the nuclear and membrane containing fraction by centrifugation at 400 × *g* for 10 min, 700 µl cell lysate was ultracentrifuged at 436,000 × *g* for 25 minutes on a 0.4 ml cushion of 25% sucrose in KCl ribosome lysis buffer using a MLA-130 rotor (Beckman, Geneva, Switzerland). Pellets were re-suspended in ribosome lysis buffer with the indicated KCl concentrations, followed by ultracentrifugation as described above. Pellets were re-suspended in KCl ribosome lysis buffer, and prepared for analysis by SDS-PAGE and Western blotting.

## Supporting Information

Figure S1 **Subcellular localization of hNaa40p-V5 in highly overexpressing cells.** A431 and HeLa cells were prepared as described in Figure 8. The same expression pattern as shown in Figure 8 (panel 1 (white arrows), and 2) was also observed in highly overexpressing cells. Scale bars are 5 µm.
(TIF)
