# Supplementary figures and images for: The Human N-Alpha-Acetyltransferase 40 (hNaa40p/hNatD) Is Conserved from Yeast and N-Terminally Acetylates Histones H2A and H4
Source: PLoS One. 2011 Sep 15;6(9):e24713. doi: 10.1371/journal.pone.0024713 (PMC3174195; doi:10.1371/journal.pone.0024713)

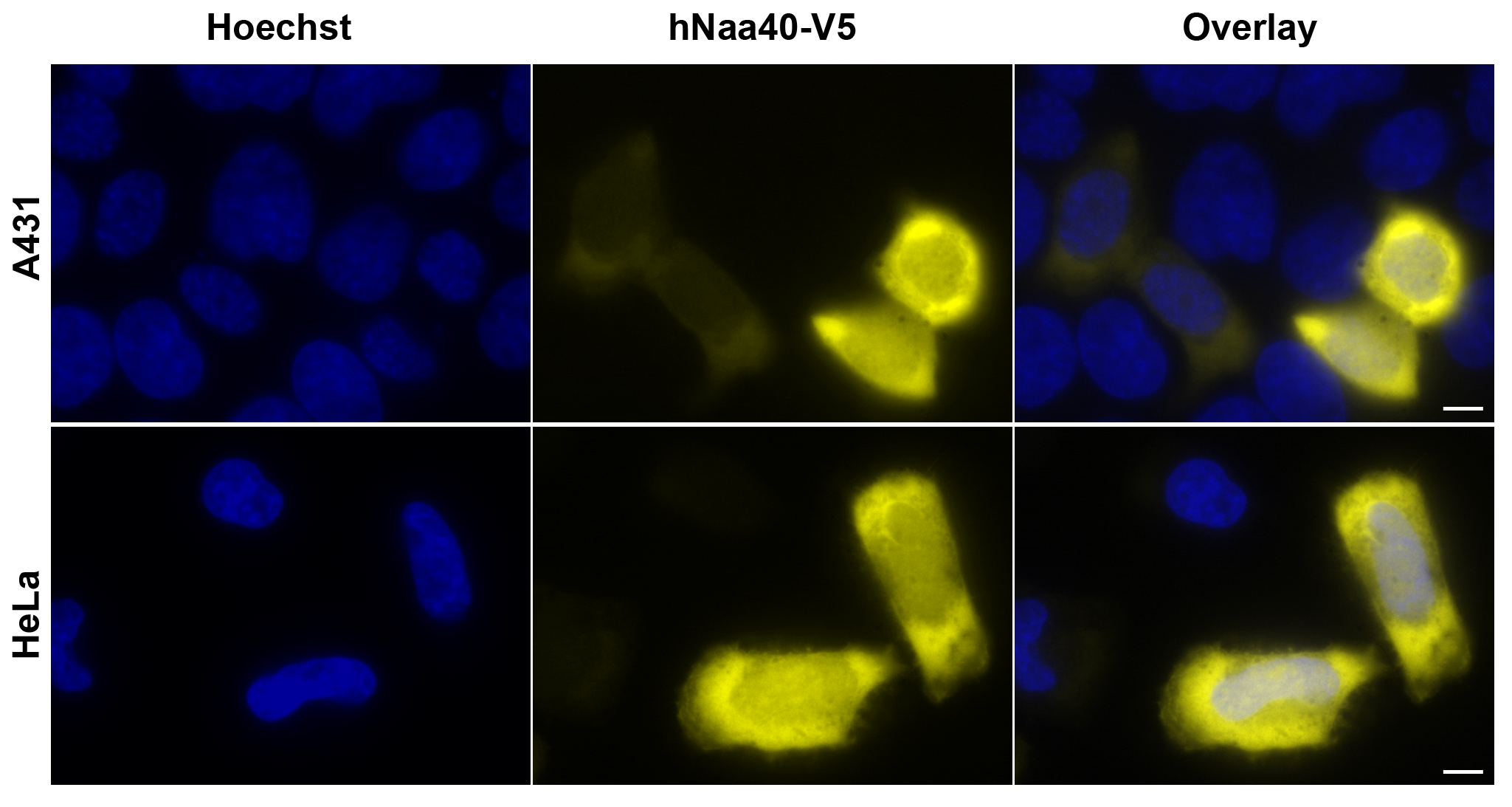

Supplement: Figure S1 — Subcellular localization of hNaa40p-V5 in highly overexpressing cells. A431 and HeLa cells were prepared as described in Figure 8. The same expression pattern as shown in Figure 8 (panel 1 (white arrows), and 2) was also observed in highly overexpressing cells. Scale bars are 5 µm. (TIF) [file pone.0024713.s001.tif]
